# Mother-to-Infant Transmission of Intestinal Bifidobacterial Strains Has an Impact on the Early Development of Vaginally Delivered Infant's Microbiota

**DOI:** 10.1371/journal.pone.0078331

**Published:** 2013-11-14

**Authors:** Hiroshi Makino, Akira Kushiro, Eiji Ishikawa, Hiroyuki Kubota, Agata Gawad, Takafumi Sakai, Kenji Oishi, Rocio Martin, Kaouther Ben-Amor, Jan Knol, Ryuichiro Tanaka

**Affiliations:** 1 Yakult Central Institute for Microbiological Research, Tokyo, Japan; 2 Yakult Honsha European Research Center for Microbiology, ESV, Gent-Zwijnaarde, Belgium; 3 Danone Research, Centre for Specialised Nutrition, Utrecht, The Netherlands; 4 Danone Research, Centre for Specialised Nutrition, Singapore; 5 Laboratory of Microbiology, Wageningen University, Wageningen, The Netherlands; Instutite of Agrochemistry and Food Technology, Spain

## Abstract

**Objectives:**

*Bifidobacterium* species are one of the major components of the infant's intestine microbiota. Colonization with bifidobacteria in early infancy is suggested to be important for health in later life. However, information remains limited regarding the source of these microbes. Here, we investigated whether specific strains of bifidobacteria in the maternal intestinal flora are transmitted to their infant's intestine.

**Materials and Methods:**

Fecal samples were collected from healthy 17 mother and infant pairs (Vaginal delivery: 12; Cesarean section delivery: 5). Mother's feces were collected twice before delivery. Infant's feces were collected at 0 (meconium), 3, 7, 30, 90 days after birth. Bifidobacteria isolated from feces were genotyped by multilocus sequencing typing, and the transitions of bifidobacteria counts in infant's feces were analyzed by quantitative real-time PCR.

**Results:**

Stains belonging to Bifidobacterium adolescentis, Bifidobacterium bifidum, Bifidobacterium catenulatum, Bifidobacterium longum subsp. longum, and Bifidobacterium pseudocatenulatum, were identified to be monophyletic between mother's and infant's intestine. Eleven out of 12 vaginal delivered infants carried at least one monophyletic strain. The bifidobacterial counts of the species to which the monophyletic strains belong, increased predominantly in the infant's intestine within 3 days after birth. Among infants delivered by C-section, monophyletic strains were not observed. Moreover, the bifidobacterial counts were significantly lower than the vaginal delivered infants until 7 days of age.

**Conclusions:**

Among infants born vaginally, several Bifidobacterium strains transmit from the mother and colonize the infant's intestine shortly after birth. Our data suggest that the mother's intestine is an important source for the vaginal delivered infant's intestinal microbiota.

## Introduction

The intestinal microbiota is a complex ecosystem with extensive metabolic activity, accounting for 10^11^ to 10^12^ bacteria per gram of feces and comprising more than 1000 bacterial species [1]–[3]. It has been recognized to play an important role in the health and disease of the host [4]–[8]. The gastrointestinal tract of newborns is suggested to rapidly become colonized with bacteria while in the womb and after birth, starting with facultative aerobes such as the *Enterobacteriaceae* and followed by anaerobic bacteria such as *Bifidobacterium* [9]–[12]. The source of these intestinal microbes is of continued interest, because increasing evidence suggests that the initial intestinal colonization provides a vast microbial stimulus leading to profound changes in the gut development and defense system [13], [14]. Colonization with bifidobacteria seems to play a crucial role in protecting the host against pathogenic bacteria, contributing to the priming of the mucosal immune system and consequently protecting against the susceptibility to diverse diseases later in life [15]–[17]. However, little information is available regarding the source of these intestinal microbes, although they are hypothesized to be derived from the maternal intestinal microbiota, diet, and the environment [18]–[20].

In a previous study, it was demonstrated that strains originated from the maternal gut were transmitted to the infant's intestine [21]. The transmitted strains were found in the first feces (meconium) of the infant and at days 3, 7, 30, and 90 after birth, indicating that these organisms stably colonize the infant's intestine soon after birth. This mother-to-infant transmission study was focused on one species, *Bifidobacterium longum* subsp. *longum*, one of the predominant bifidobacterial species in the intestine of adults and infants [22]. Moreover, the investigated subjects were all mother and infant pairs who gave birth by vaginal delivery, and infants delivered by Cesarean section (C-section) were not included in the study. Although cesarean delivered infants has been suggested to be initially colonized by bacteria from the environment, hospital staff and/or other neonates [23], the influence on the occurrence of mother-to-infant transmission among the differences in mode of delivery was not examined.

Because little is known about the vertical transmission of bifidobacteria from mothers to their newborn infants, the aim of this study was to verify whether mother-to-infant transmission is occurring among several different *Bifidobacterium* species in the infant's gut microbiota. Furthermore, we have undertaken to investigate whether the difference in mode of delivery influences the mother-to-infant transmission. For this purpose, we have collected fecal samples at different times from vaginal delivered and cesarean delivered mother and infant pairs, isolated *Bifidobacterium* strains from the fecal samples and identified the strains similarity within mother and infant, and analyzed the transition of the numbers of bifidobacteria.

## Materials and Methods

### Collection of the samples

Samples of feces were collected aseptically from 17 mother and infant pairs (vaginal delivery: 12 pairs; cesarean delivery: 5 pairs) (see table S1). Fecal samples were taken from mothers twice (at least 1 week apart) before delivery (see table S2), and those from infants were collected at 0 (meconium), 3, 7, 30, and 90 days of age. All mothers were healthy and had full-term pregnancies and all of the infants were exclusively breast-fed for at least 2 months. These subjects belong to a larger observational study conducted in Belgium (protocol number ISRCTN66704989).

Participants were given a sterile glass tube containing 6 mL of anaerobic transport medium [content in 1 L: 0.225 g KH_2_PO_4_, 0.225 g K_2_HPO_4_, 0.45 g NaCl; 0.225 g (NH_4_)_2_SO_4_, 0.0225 g CaCl_2_, 0.0225 g MgSO_4_, 0.5 g L-cysteine hydrochloride, 0.001 g resazurin, 0.5 g agar, 10 g Lab-Lemco powder (Oxoid Ltd., Basingstoke, England), 100 mL glycerol; and 2.1 mL 8% Na_2_CO_3_] [24], and an empty tube for the qPCR reaction. They placed a spoonful of fecal sample (approximately 0.5 g) into each collection tube immediately after defecating. Samples were kept at 4°C until delivery to the laboratory, which occurred within 72 h after collection.

This study was conducted in accordance with ethical principles that originated from the Declaration of Helsinki, guidelines of good clinical practice, and applicable regulatory requirements. The ethics committee of the hospital network of Antwerp (Ziekenhuisnetwerk Antwerpen) approved the study, and informed written consent was obtained from the mothers.

### Isolation and identification of *Bifidobacterium* species

Fecal samples in anaerobic transport medium were homogenized by vortex mixer to create fecal suspensions, which were serially diluted with phosphate-buffered saline (pH 7.2), inoculated onto TOS propionate agar (Yakult Pharmaceutical Industry Co., Ltd., Tokyo, Japan) containing 50 µg/mL mupirocin (TOS-M agar), and incubated anaerobically at 37°C for 72 h. From each sample, 2 to 6 colonies showing different morphologies on the medium were isolated for subsequent analyses.

DNA was extracted from all pure cultures of the colonies sampled from the agar plates as described [25], with slight modification. Briefly, all isolated strains were cultured anaerobically in TOS-M broth for 48 h at 37°C. Cell pellets were collected from 1.0 mL of each culture by centrifugation (20,000× *g*, 5 min, 4°C). Pellets were resuspended in 250 µL extraction buffer (100 mM Tris·HCl, 40 mM EDTA; pH 9.0). Glass beads (diameter, 0.1 mm; 700 mg), 500 µL phenol, and 50 µL 10% sodium dodecyl sulfate were added to each resuspension, and the mixture was vortexed vigorously for 30 s by using a FastPrep-24 (M.P. Biomedicals, Irvine, CA) at power level of 6.5. After vortexing, 150 µL 3 M sodium acetate was added to each sample, which then was cooled on ice for 5 min. After centrifugation (20,000× *g*, 8 min, 4°C), the supernatant was collected, and DNA was precipitated with isopropanol. Finally, each DNA pellet was diluted in 100 µL TE buffer (10 mM Tris·HCl, 1 mM EDTA; pH 8.0).

Isolates were identified at the species level through polymerase chain reaction (PCR) sequencing of the 16S rRNA gene by using the universal primers 8F (5′-AGAGTTTGATCCTGGCTCAG-3′) and 1492R (5′-ACGGCTACCTTGTTACGACTT-3′) [26]. PCR was carried out in 25-µL reaction, containing 10 mM Tris·HCl (pH 8.3), 50 mM KCl, 1.5 mM MgCl_2_, 200 µM each dNTP, 0.5 U *Taq* DNA polymerase (Takara, Shiga, Japan), 0.4 µM of each respective primer, and 10 ng DNA template. The PCR amplification program consisted of an initial heating step at 94°C for 2 min; 32 cycles of 94°C for 20 s, 55°C for 20 s, and 72°C for 20 s; and a final extension step at 72°C for 3 min. All amplifications were performed on a C1000™ Thermal Cycler (Bio-Rad, Hercules, CA). Amplicons were purified by using ExoSAP-IT (USB, Cleveland, OH) and sequenced by using the primers 8F and 520R (5′-ACCGCGGCTGCTGGC-3′) [27], and BigDye Terminator v1.1 chemistry (Life Technologies, Carlsbad, CA) on a 3130xl Genetic Analyzer (Life Technologies). The resulting sequences were used to search sequences deposited in the EMBL database by using the BLAST algorithm (http://blast.ddbj.nig.ac.jp/blast/blastn?lang=en), and the identities of the isolates were determined on the basis of the highest scores. Isolated strains that belonged to *Bifidobacterium catenulatum/Bifidobacterium pseudocatenulatum* continuum were further identified by PCR sequencing using primers BClpC-F (5′-ATCGCSGARACBATYGAGA-3′) and BClpC-R (5′-ATRATGCGCTTGTGCARYT-3′) [28] for the ClpC ATPase (*clpC*) gene, and primers B11 (5′-GTSCAYGARGGYCTSAAGAA-3′) and B12 (5′-CCRTCCTGGCCRACCTTGT-3′) [29] for the heat-shock protein 60 (*hsp60*) gene (see figure S1 for the identification of *B. catenulatum/B. pseudocatenulatum* continuum), according to the methodology described above.

### Multilocus sequencing typing (MLST) analysis

For the species belonging to *Bifidobacterium adolescentis*, *Bifidobacterium bifidum*, *B. catenulatum*, and *B. pseudocatenulatum* isolates, multilocus sequencing typing (MLST) analysis was performed using the seven housekeeping genes (*clpC*, *fusA*, *gyrB*, *ileS*, *purF*, *rplB*, *rpoB*) previously described in the MLST protocol by Delétoile *et al.* [30], with slight modification. Isolates that belonged to *B. longum* subsp. *longum* were analyzed by using these seven housekeeping genes (*clpC*, *dnaG*, *dnaJ*, *fusA*, *gyrB*, *purF*, *rpoB*) [21]. Each 25-µL reaction contained 1× PCR buffer, 200 µM dNTPs, 2 mM MgCl_2_, 0.4 µM of each primer (Table 1), 10 µL GC-RICH solution, 2 U Fast *Taq* polymerase (Roche, Basel, Switzerland), and 10 ng template DNA. The PCR amplification program consisted of an initial heating step at 95°C for 5 min; 30 cycles of 95°C for 30 s, 57°C for 30 s, and 72°C for 1 min; and a final extension step at 72°C for 10 min. For compensating unavailable nucleotide sequence for *rplB* gene, the following primer set was used; forward: 5′-TGGTGCTCAGGCTGATATCA-3′, reverse: 5′ -TAAGCGCCATCCTTGGCG-3′. Products were sequenced as described above.

**Table 1:** Primer sets for MLST analysis.

Target species	Gene	Primer	Sequence (5′–3′)	Amplicon size (bp)
*B. adolescentis*	*clpC*	ClpC-uni	GAGTACCGCAAGTACATCGAG	600
*B. bifidum*		ClpC-rev	CATCCTCATCGTCGAACAGGAAC	
*B. catenulatum*	*fusA*	fusAB3	ATCGGCATCATGGCYCACATYGAT	666
*B. pseudocatenulatum*		fusAB4	CCAGCATCGGCTGMACRCCCTT	
	*gyrB*	gyrBB3	AGCTGCACGCBGGCGGCAAGTTCG	627
		gyrBB4	GTTGCCGAGCTTGGTCTTGGTCTG	
	*ileS*	ileSB3	ATCCCGCGYTACCAGACSATG	489
		ileSB4	CGGTGTCGACGTAGTCGGCG	
	*purF*	PurF-uni	CATTCGAACTCCGACACCGA	591
		PurF-rev	GTGGGGTAGTCGCCGTTG	
	*rplB*	rplBB3	GGACAAGGACGGCRTSCCSGCCAA	357
		rplBB4	ACGACCRCCGTGCGGGTGRTCGAC	
	*rpoB*	rpoBB3	GGCGAGCTGATCCAGAACCA	501
		rpoBB4	GCATCCTCGTAGTTGTASCC	
*B. longum* subsp. *longum*	*clpC*	Bilon-clpC-F	CCTGAAGAAGGTGCTGAAGG	563
		Bilon-clpC-R	TTCTCCTGCTTGTCGCGCAGT	
	*dnaG*	Bilon-dnaG-F	GTTGCCGTAGATTTGGGCTTGG	449
		Bilon-dnaG-R	ATGACTTCGGTGTTCCGCAC	
	*dnaJ*	Bilon-dnaJ-F	GCTGAGCAAGAAGGAAGATCGC	421
		Bilon-dnaJ-R	TGAACTTCTTGCCGTCCACGG	
	*fusA*	Bilon-fusA-F	CACCATCAAGGAGAAGCTGG	536
		Bilon-fusA-R	ACGAGCTTGCCGTAGAACG	
	*gyrB*	Bilon-gyrB-if1	AAGTGCGCCGTCAGGGCTT	473
		Bilon-gyrB-R	GTGTTCGCGAAGGTGTGCAC	
	*purF*	Bilon-purF-if1	ATGGCGGTTTCGCCTACC	501
		Bilon-purF-ir1	AGAGAGCTTCATACGCACAC	
	*rpoB*	Bilon-rpoB-F	AGACCGACAGCTTCGATTGG	575
		Bilon-rpoB-R	AACACGATGGCGGACTGCTT	

Primers targeting species belonging to *B. adolescentis*, *B. bifidum*, *B. catenulatum*, *B. pseudocatenulatum* were designed by Delétoile *et al.* [30], and *B. longum* subsp. *longum* primers were designed by Makino *et al.* [21].

BioNumerics software version 6.6 (Applied-Maths, Sint-Martens-Latem, Belgium) was used to perform all phylogenetic analyses. For MLST data, the sequences obtained for the seven housekeeping genes were aligned and compared. Each distinct gene sequence was assigned an allele number, and each unique combination of seven allele numbers was assigned a sequence type (ST). Based on allelic profiles, cluster analysis of the categorical coefficient was performed using the unweighted pair group method with arithmetic means (UPGMA) algorithm implemented in BioNumerics. This categorical parameter implies that the same weight is given to any multistate character at each locus, whatever the repeat number is [31]. Nucleotide sequences of MLST loci were deposited in GenBank/EMBL/DDBJ under accession numbers AB845863 to AB845966 and AB845704 (*B. adolescentis*), AB845967 to AB846141 (*B. bifidum*), AB846142 to AB846190 (*B. catenulatum*), AB846233 to AB846582 (*B. longum* subsp. *longum*), and AB846191 to AB846232 (*B. pseudocatenulatum*).

### Quantitative real-time PCR (qPCR) assays

Quantitative real-time PCR (qPCR) was used to determine the population sizes of the predominant and subdominant bifidobacteria groups, as described by Matsuki *et al.* [32]. First, fecal samples (20 mg) stocked in the empty tubes were washed 3 times by suspending them in 1 mL of phosphate-buffered saline (PBS) (Nissui Pharmaceutical Co. Ltd., Tokyo, Japan) to extract DNA. The thawed sample was mixed with 250 µL of extraction buffer and 50 µL of 10% sodium dodecyl sulfate. Three hundred mg of glass beads (diameter, 0.1 mm) and 500 µL of TE-saturated phenol (Sigma-Aldrich, St. Louis, MO) were added to the suspension, and the mixture was vortexed vigorously for 30 s using a FastPrep-24 at a power level of 5.0. Phenol-chloroform extractions were performed, and 250 µL of the supernatant was subjected to isopropanol precipitation. Finally, the DNA was suspended in 1 mL of TE buffer.

PCR amplification and detection were performed with a 7900HT Fast Real-Time PCR System and SDS software (version 4.0) (Life Technologies). The reaction mixture (10 µL) was composed of 10 mM Tris-HCl, pH 8.3; 50 mM KCl; 1.5 mM MgCl_2_; 200 µM of each dNTP; 1∶75,000 dilution of SYBR Green I (Molecular Probes, Eugene, OR); 11 ng TaqStart antibody (ClonTech, Palo Alto, CA); 0.05 U Taq DNA polymerase (Takara); 0.25 µM of each of the specific primers (Table 2); and 1 µL of ×10, ×100, or ×1000 diluted template DNA. The amplification program consisted of one cycle at 94°C for 5 min; 40 cycles at 94°C for 20 s, 55°C for 20 s, and 72°C for 50 s; and finally one cycle at 94°C for 15 s. Fluorescent products were verified at the last step of each cycle. The bacterial count was estimated from the slope of the standard curve, generated by using the following type strains: *B. adolescentis* ATCC 15703^T^ (for the *B*. *adolescentis* group), *B. bifidum* ATCC 29521^T^ (for *B. bifidum*), *B. breve* ATCC 15700^T^ (for *B. breve*), *B. dentium* ATCC 27534^T^ (for *B. dentium*), *B. longum* subsp. *infantis* ATCC 15697^T^ (for *B. longum* subsp. *infantis*), *B. longum* subsp. *longum* ATCC 15707^T^ (for *B. longum* subsp. *longum*), *B. pseudocatenulatum* JCM 1200^T^ (for the *B. catenulatum* group). The total bifidobacteria was expressed as the sum of the counts of the measured 7 predominant bifidobacterial species. For values less than the qPCR detection limit (10^6^ cells/g of feces), “detection limit/√2” [34] was used as a complement value. Statistical analysis was performed to compare the total bifidobacterial population between vaginal delivered infants and cesarean delivered infants, with the Wilcoxon rank-sum test (SAS version 8.02, SAS Institute Inc., Cary, NC).

**Table 2:** 16S rRNA gene targeted primer sets used for qPCR.

Target	Primer	Sequence (5′ - 3′)	Reference
*Bifidobacterium adolescentis* group^a^	BiADOg-1a	CTCCAGTTGGATGCATGTC	[32]
	BiADOg-1b	TCCAGTTGACCGCATGGT	
	BiADO-2	CGAAGGCTTGCTCCCAGT	
*Bifidobacterium bifidum*	BiBIF-1	CCACATGATCGCATGTGATTG	[33]
	BiBIF-2	CCGAAGGCTTGCTCCCAAA	
*Bifidobacterium breve*	BiBRE-1	CCGGATGCTCCATCACAC	[33]
	BiBRE-2	ACAAAGTGCCTTGCTCCCT	
*Bifidobacterium catenulatum* group^b^	BiCATg-1	CGGATGCTCCGACTCCT	[33]
	BiCATg-2	CGAAGGCTTGCTCCCGAT	
*Bifidobacterium dentium*	BiDEN-1	ATCCCGGGGGTTCGCCT	[22]
	BiDEN-2	GAAGGGCTTGCTCCCGA	
*Bifidobacterium longum* subsp. *infantis*	BiINF-1	TTCCAGTTGATCGCATGGTC	[22]
	BiINF-2	GGAAACCCCATCTCTGGGAT	
*Bifidobacterium longum* subsp. *longum*	BiLON-1	TTCCAGTTGATCGCATGGTC	[22]
	BiLON-2	GGGAAGCCGTATCTCTACGA	

a The *B. adolescentis* group consists of *B. adolescentis* genotypes A and B.

b The B. catenulatum group consists of B. catenulatum and B. pseudocatenulatum.

## Results

### Identification of *Bifidobacterium* species obtained from the feces of mothers and infants

In total, 273 bifidobacterial isolates were obtained from 17 mother-infant pairs (vaginal delivered pairs: 211 isolates; cesarean delivered pairs: 62 isolates). Among the 12 mother-infant pairs who gave birth by vaginal delivery, *B. adolescentis*, *B. bifidum*, *B. catenulatum*, *B. longum* subsp. *longum*, and *B. pseudocatenulatum* were the five *Bifidobacterium* species that were obtained from the feces of both mothers and infants (see table S3). *B. adolescentis* was isolated from both mother and infant from 3 families. Likewise, *B. bifidum* was isolated from 7 families (including one family which gave birth to twins), *B. catenulatum* from 2 families, *B. longum* subsp. *longum* from 5 families, and *B. pseudocatenulatum* from 2 families.

On the other hand, *B. longum* subsp. *longum*, was the only species that was obtained from the feces of both mothers and infants among the 5 mother-infant pairs who gave birth by C-section.

### Comparison of MLST profiles of *Bifidobacterium* strains isolated from both members of a mother-infant pair

The 273 isolates (*B. longum* subsp. *longum*, 153 isolates; *B. bifidum*, 64 isolates; *B. adolescentis*, 32 isolates; *B. catenulatum*, 15 isolates; *B. pseudocatenulatum*, 9 isolates) were determined by the sequences of the seven loci. Isolates that had the same STs, and were obtained from the same source and at the same sampling point were defined as identical strains; otherwise, they were identified as individual strains. Consequently, among the 273 *Bifidobacterium* isolates, 103 individual strains were identified (*B. longum* subsp. *longum*, 50 strains; *B. bifidum*, 25 strains; *B. adolescentis*, 15 strains; *B. catenulatum*, 7 strains; *B. pseudocatenulatum*, 6 strains). An unweighted pair group method with arithmetic mean (UPGMA) dendrogram was generated based on the MLST profiles for all 103 individual stains.

### (i) Vaginal delivered mother-infant pair

**Figure 1:**
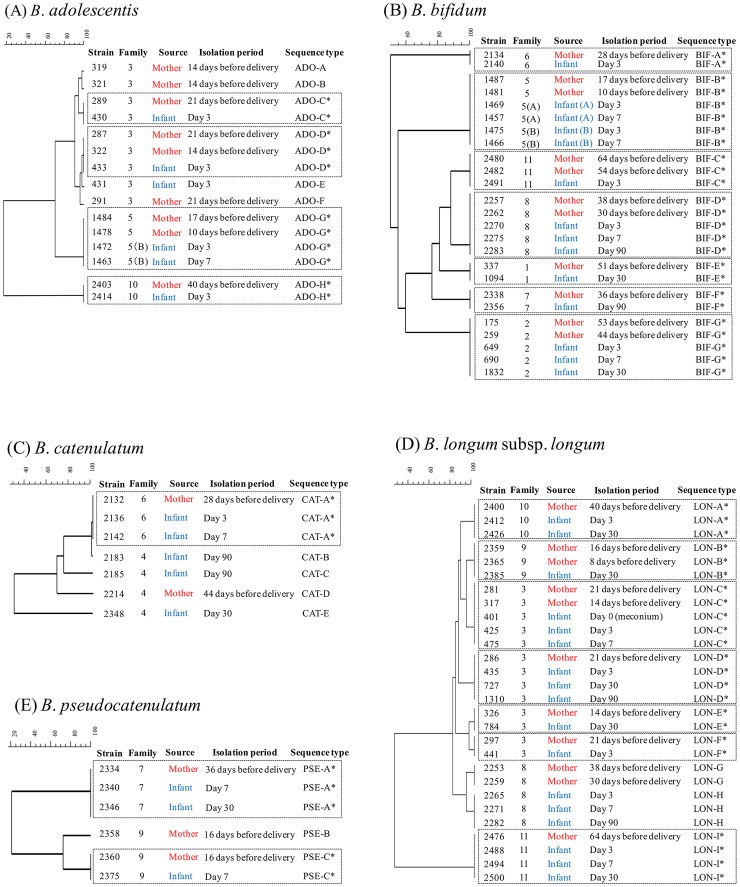
Comparison of MLST profiles of strains obtained from 12 vaginal delivered mother-infant pairs. Dendrogram of (A) 15 individual strains of *B. adolescentis* (ADO), (B) 25 individual *B. bifidum* strains (BIF), (C) 7 individual *B. catenulatum* strains (CAT), (D) 28 individual *B. longum* subsp. *longum* strains (LON), (E) 6 individual *B. pseudocatenulatum* strains (PSE) were all generated by a multi-scale setting for comparison and UPGMA for clustering. ^*^ Isolates from both members of a mother–infant pair and showing the same MLST profile within a given cluster.

Among the 12 vaginal delivered mother-infant pairs, four strains of *B. adolescentis* (STs: ADO-C, ADO-D, ADO-G, ADO-H; Figure 1A), seven of *B. bifidum* (STs: BIF-A to BIF-G; Figure 1B), one of *B. catenulatum* (ST: CAT-A; Figure 1C), seven of *B. longum* subsp. *longum* (STs: LON-A, LON-B, LON-C, LON-D, LON-E, LON-F, LON-I; Figure 1D), and two of *B. pseudocatenulatum* (STs: PSE-A and PSE-C; Figure 1E) were identified as monophyletic between mothers and infants. These mother-infant monophyletic strains were isolated from the mother's feces before delivery at different times, and also from the infant's feces at different time. The mother-infant monophyletic strains such as STs BIF-D, BIF-G, LON-A, LON-D, and LON-I, were isolated not only from infants at age day 3 but also from day 30 and/or 90 (Figure 1B and 1D).

Family no. 5, which gave birth to twins, an identical mother-infant monophyletic *B. bifidum* strain was found from both of the twins. The monophyletic strain (ST: BIF-B; Figure 1B) was isolated from the mother's feces 17 and 10 days before delivery and also on days 3 and 7 from both of the twin infants.

### (ii) Cesarean delivered mother-infant pair

**Figure 2:**
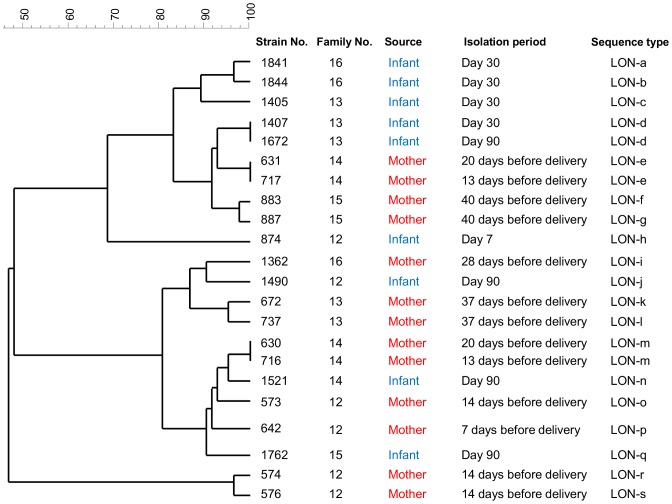
Dendrogram of 22 individual *B. longum* subsp. *longum* isolates from 5 cesarean delivered mother-infant pairs. All dendrogram were generated by a multi-scale setting for comparison and UPGMA for clustering.

Among the 5 mother-infant pairs which gave delivery by C-section, none of the strains were identified as monophyletic between mothers and infants (Figure 2). Two strains were monophyletic within the same mother, collected at different sampling period (STs: LON-e and LON-m), and one strain was monophyletic only within the same infant (ST: LON-d).

### Diversity among the mother-infant monophyletic *Bifidobacterium* species

Mother-infant monophyletic strains were found among all five *Bifidobacterium* species, which were obtained from fecal samples of both mother and infant (Table 3). These strains were obtained only from families which gave birth by vaginal delivery. Eleven out of 12 mother-infant pairs yielded at least one mother-infant monophyletic *Bifidobacterium* strain. Moreover, strains from two different species were monophyletic between 7 mothers and their children (nos. 3, 5, 6, 7, 9, 10, 11).

**Table 3:** Detection of mother-infant monophyletic *Bifidobacterium* strains among 16 families.

		Mother–infant monophyletic *Bifidobacterium* strains obtained from mother-infant pair				
Mode of delivery	Family No.	*B. adolescentis*	*B. bifidum*	*B. catenulatum*	*B. longum* subsp. *longum*	*B. pseudocatenulatum*
Vaginal delivery	1		•			
	2		•			
	3	•			•	
	4					
	5 (A)		•			
	5 (B)	•	•			
	6		•			•
	7		•			•
	8		•			
	9			•	•	
	10	•			•	
	11		•		•	
Cesarean delivery	12					
	13					
	14					
	15					
	16					

“•” represents that at least one mother-infant monophyletic *Bifidobacterium* strain was isolated from the indicated family. Mother no. 5 gave birth to twins (A, B).

### Transitions of the bifidobacteria population within the infant's intestine

**Figure 3:**
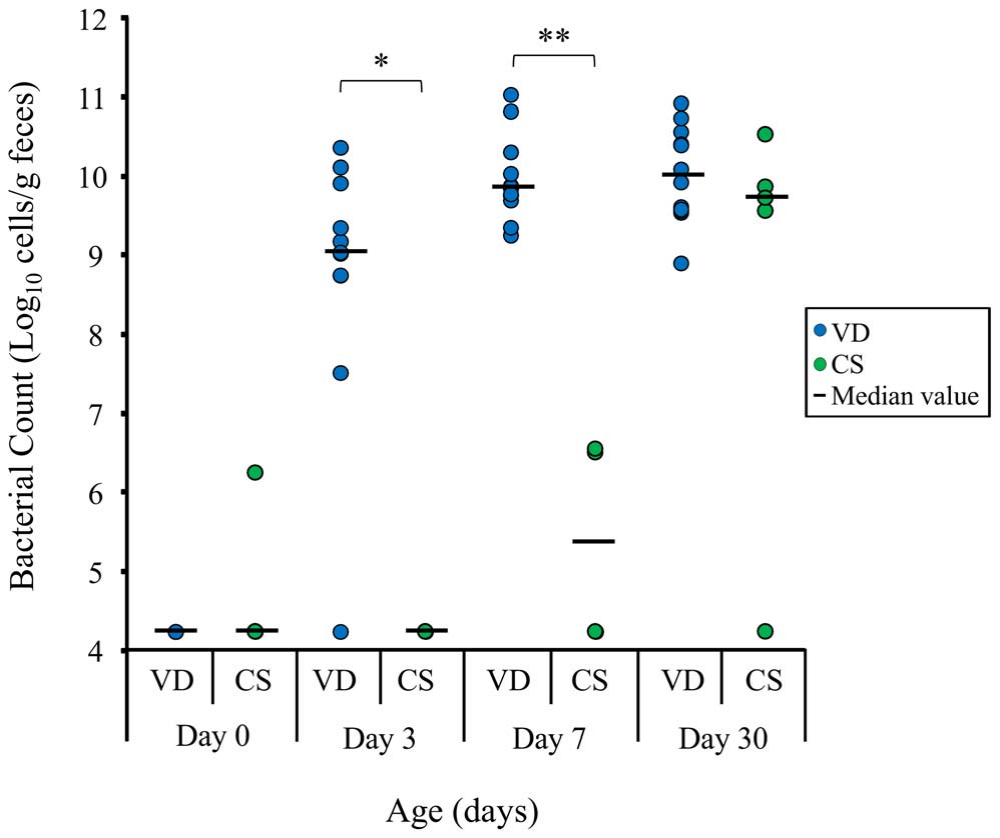
The total bifidobacteria detected in 17 infant's feces from 0 to 7 days of age. Numbers of vaginal delivered (VD) infants were 12, infants delivered by C-section (CS) were 5. Bars represent the median values respectively. For values less than the qPCR detection limit (10^6^ cells/g of feces), “detection limit/√2” [34] was used as a complement value. Stars indicate significant differences between VD infants and CD infants (*P<0.05; **P<0.01).

The population sizes of the total bifidobacteria and the predominant/subdominant *Bifidobacterium* species were analyzed by qPCR. For the vaginally delivered infants, the total bifidobacterial counts showed an increase from about 10^4^ cells/g of feces, which is the complement value for values less than the qPCR detection limit (10^6^ cells/g of feces), to about 10^10^ cells/g of feces during the 7 days after birth (Figure 3). On the other hand, the bifidobacteria count of the fecal samples from for the infants delivered by C-section was below 10^6^ cells/g of feces at all the time points.

**Figure 4:**
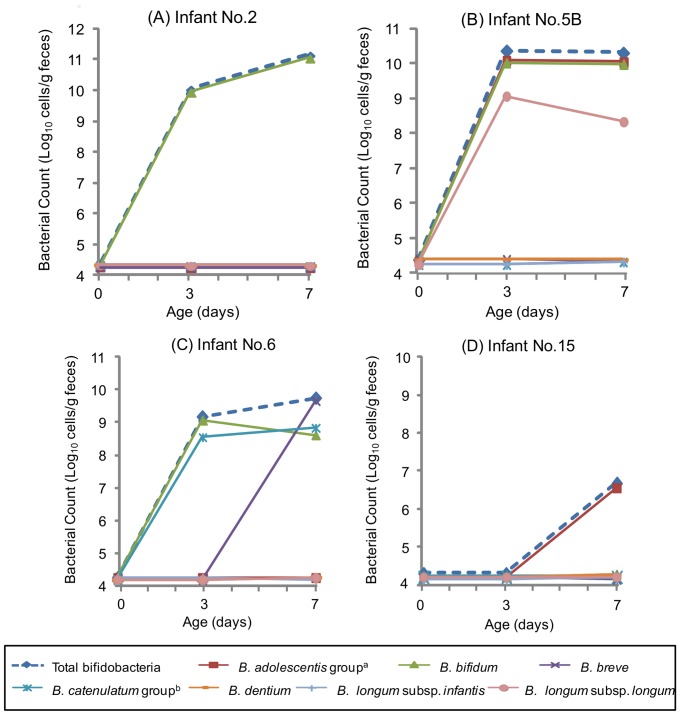
The numbers of bifidobacteria in infants from 0 day to 7 days after birth. Infant no.^a^ The *B. adolescentis* group consists of *B. adolescentis* genotypes A and B. ^b^ The *B. catenulatum* group consists of *B. catenulatum* and *B. pseudocatenulatum*.

Confirming the transition and stability of bifidobacteria in infants individually, vaginal delivered infant such as no. 2, the *B. bifidum* counts increased from bellow the detection limit to 10^11^ cells/g of feces from 0 to 7 days of age. This bacterial count showed equivalent numbers with the total bifidobacteria, indicating that this species was the most and the only predominant bifidobacteria during early infancy (Figure 4A). The monophyletic *B. bifidum* strains were found to within the no. 2 mother-infant pair (Table 3). Likewise, the two most predominant species for vaginal delivered infant no. 5B (*B. adolescentis*, *B. bifidum*) and no. 6 (*B. bifidum*, *B. catenulatum*) were the species which were transmitted from each of their mother (Figure 4, Table 3). Such increase in numbers of the bifidobacterial species, to which the monophyletic strains belong, was confirmed among 11 out of 12 vaginally born infants. On the other hand, infant delivered by C-section such as no. 15, bifidobacteria was rarely detected during the first weeks of life, as the total bifidobacteria was below the detection limit until 3 days of age and increased only up to 10^6.5^ cells/g at 7days after birth (Figure 4D). The other four infants delivered by C-section have also showed a similar transition as bifidobacteria being rarely detected during the early infancy.

## Discussion

Among the five Bifidobacterium species (B. adolescentis, B. bifidum, B. catenulatum, B. longum subsp. longum, B. pseudocatenulatum), we identified four strains of B. adolescentis, seven of B. bifidum, one of B. catenulatum, seven of B. longum subsp. longum, and two of B. pseudocatenulatum that were monophyletic between mothers and infants (Figure 1A–1E, Table 3). These findings confirm that mother-to-infant transmission occurs among several Bifidobacterium species. Moreover, two strains from different species were monophyletic in several families (Table 3), confirming that mother-to-infant transmission occurs parallel among several Bifidobacterium species within a single family. These results suggest that other commensal bacterial species might also transmit from the mother and colonize the intestine of the infant soon after birth.

Mother-infant monophyletic *Bifidobacterium* strains were obtained from 11 out of 12 vaginally born infants (Table 3). On the other hand, these monophyletic strains were not observed among any of the 5 infants delivered by C-section (Figure 2), verifying that mother-to-infant transmission occurrence was confirmed only among the vaginally born infants. This study relies on cultivation technique. This hampers the ability to evaluate the exact frequency of mother-to-infant transmission occurrence. However, if the mother-to-infant transmission occurs with the same frequency as the mother-infant pairs who gave birth by vaginal delivery, it is not surprising to find mother-infant monophyletic strains from at least 1 out of 5 infants delivered by C-section. These results suggest that the delivery mode may influence the occurrence of mother-to-infant transmission.

The bifidobacterial counts of the species to which the monophyletic strains belong, increased predominantly in the vaginal delivered infant's intestine within 3 days after birth (Figure 4A–4C). On the other hand, among infants that were born by C-section, the number of the total bifidobacteria was significantly lower than that in the vaginally delivered infants until 7 days of age (Figure 3; Day 3: *P* = 0.0223, Day 7: *P* = 0.0042). It has been proposed that the intestinal colonization of bifidobacteria starts faster among vaginal delivered infants than infants delivered by C-section [35], [36]. Our results suggest that among infants born vaginally, the bifidobacterial increase within few days after birth may have a correlation with the occurrence of the mother-to-infant transmission. Moreover, since mother-infant monophyletic strains were not observed in the infants delivered by C-section, the maternal strains might have been transmitted during the transit through the birth canal. Our results confirmed that strains in the pregnant mother's intestine transmit to their infant's intestine. However, the relationship of the bacterial strains between the vagina and the intestine was not analyzed. In some studies, it have been shown that there is a certain degree of correspondence between the rectal and the vaginal microbiota, suggesting that the intestine can be a source for the vaginal microbiota [37], [38]. Breast milk and oral samples were also not obtained for this study. Further strain-level investigation among these sources will aim to clarify the route of these mother-to-infant transmission strains.

The environment such as hospital staff or other neonates are also suggested to be one of the sources of the infant's microbiota; strains being transmitted horizontally to the newborn's intestine after birth [23]. In our study, nine infants (nos. 1, 2, 3, 4, 6, 9, 12, 13, 15) were born in the same hospital (see table S1). Among these infants, monophyletic strains among different infants were not observed. Likewise, none of the strains were identified as monophyletic among the other infants (nos. 5A, 5B, 8, 10; 7, 14) which were born in the same hospital. Thus, we did not find evidences which can show the occurrence of horizontal transmission for bifidobacteria.

Several studies suggest that in addition to environment, diet, and delivery mode, host genetic may also be a key factor that influences the composition of the intestinal microbiota [39], [40]. In a previous study, mother-infant monophyletic *B. longum* subsp. *longum* strains were specific to each family and were not found in the clusters derived from other families [21]. Similarly, in this study, mother-infant monophyletic strains among all five of the *Bifidobacterium* species that we evaluated formed an individual cluster for each family, suggesting that each family has its own unique bifidobacterial group, which is transmitted from mother to infant. Furthermore, the vertical transfer concept is strengthened by the fact that *Bifidobacterium* strains from the mother (family 5) can seed the intestine of both twin progeny (Figure 1B, Table 3). This is in agreement with previous studies that each family harbors his or her own set of *Lactobacillus* and *Bifidobacterium* species [41].

Interestingly, not all mother-infant monophyletic *Bifidobacterium* strains were isolated throughout the sampling period. Mother-infant monophyletic *B. bifidum* and *B. longum* subsp. *longum* strains were isolated from the infant fecal samples up to 90 days after birth (Figure 1B and 1D). The bacterial count also showed that these species were predominates from day 3 until day 90 of age (see figure S2). On the other hand, monophyletic strains belonging to *B. adolescentis* and *B. catenulatum* were not found in the infant's fecal samples after 7 days of age (Figure 1A and 1C). Moreover, although several vaginal delivered infants harbored these species as high as 10^10^ cells/g of feces, these species were basically not the dominant species in the vaginal delivered infant's intestine during the early infancy as the median value showing bellow 10^6.4^ cells/g of feces (see figure S2). These results suggest that some bifidobacterial species are predominantly proliferating and colonizing in the vaginal delivered infant's gut. All infants in our study were exclusively breastfed for at least 2 months. It is widely known that human milk contains a wide variety of complex oligosaccharides (HMO) that selectively stimulate the growth of specific bifidobacterial species [42], [43]. Typical infant species such as *B. bifidum* and *B. longum* are known to effectively degrade HMOs [44]–[48], while adult type bifidobacteria such as *B. adolescentis* are less efficient in degrading HMOs [46], [48]. Therefore, breastfeeding and the HMOs present may be one of the key factors to explain why, while infants acquire a wide spectrum of bifidobacterial species from the mother, species that are able to degrade HMOs become one of the predominant colonizers in the infant's gut.

*B. breve* is known to be one of the predominant species in the infant's intestinal microbiota, and is able to utilize HMOs [47], [49]. It has also been suggested that *B. breve* strains might be transferred from mother to infant [49], [50]. While it was found to be common in infant's microbiota, we could not isolate any *B. breve* strains from the mother, due to low prevalence of this bifidobacterial species in the maternal intestinal microbiota as previously described [49]. Because of this limitation of our study using cultivation technique, we were not able to assess the mother-to-infant transmission of this species, and therefore dismissing any such transmission would be questionable. Although cultivation technique cannot accurately assess the occurrence of mother-to-infant transmission, we are convinced that our results show, at a strain-specific level, that several species are transmitted from mother to infant and provide genetic data to support the hypothesis that bacteria are transmitted from mother to infant soon after birth. New techniques, such as single cell cultivation, that allows for bacterial cells isolation and characterization are rapidly developing and will allow us to expand our knowledge.

In conclusion, our results show that mothers who gave birth vaginally transmit their unique family-specific strains to their infant's intestine during early infancy. Factors like nutrition may influence the persistence of family-specific bifidobacteria strains in infants throughout the sampling period, where they become one of the predominant bifidobacteria groups during early infancy. Early bacterial colonization after birth is suggested to be crucial for the future well-being of the human host [5], [15]. In addition, bifidobacteria are believed to exert beneficial effects on the development of the mucosal immune system [16], [17]. Our data suggest that delivery mode and the mother's intestinal bifidobacteria strains are key factors in determining their infant's bifidobacterial microbiota during early infancy. Furthermore, the maintenance of a healthy and balanced intestinal microbiota during pregnancy is to be considered as an important factor to positively influence the newborn's intestinal microbiota. Further strain-level investigation will aim to clarify how mother-to-infant transmission influences other components of the intestinal microbiota during infancy.

## Supporting Information

Figure S1 **Polymorphic sites among the 24** ***B. catenulatum/B. pseudocatenulatum*** **continuum at gene** ***clpC*** **and** ***hsp60*** **.** For each gene, all discovered alleles were compared, and only polymorphic sites are shown. Numbering starts at the beginning of the aligned sequence portion of each gene.
(TIF)

Figure S2 **Bifidobacterial counts of each species in infant's feces from 0 to 90 days of age.** Bars represent the median values respectively. For values less than the qPCR detection limit (10^6^ cells/g of feces), “detection limit/√2” [34] was used as a complement value. ^a^ The *B. adolescentis* group consists of *B. adolescentis* genotypes A and B. ^b^ The *B. catenulatum* group consists of *B. catenulatum* and *B. pseudocatenulatum*.
(TIF)

Table S1 **Information of the infant (gender, gestational ages, weights, place of birth, delivery date). Mother no. 5 gave birth to twins (A, B).**
(DOCX)

Table S2 **Scheme for mother's fecal samples. Samples of feces from mothers were collected twice (at least 1 week apart) before delivery.**
(DOCX)

Table S3 **Numbers of** ***Bifidobacterium*** **strains isolated from each family and submitted for MLST analysis.** We used *Bifidobacterium* strains from 16 families (vaginal delivery: 11 families; Cesarean delivery: 5 families), isolated from mother's and infant's feces at different sampling time points. “-” represents no strains were isolated. Mother's fecal samples were collected twice before delivery (see table S2). Mother no. 5 gave birth to twins (A, B).
(DOCX)
