# Supplementary figures and images for: Mother-to-Infant Transmission of Intestinal Bifidobacterial Strains Has an Impact on the Early Development of Vaginally Delivered Infant's Microbiota
Source: PLoS One. 2013 Nov 14;8(11):e78331. doi: 10.1371/journal.pone.0078331 (PMC3828338; doi:10.1371/journal.pone.0078331)

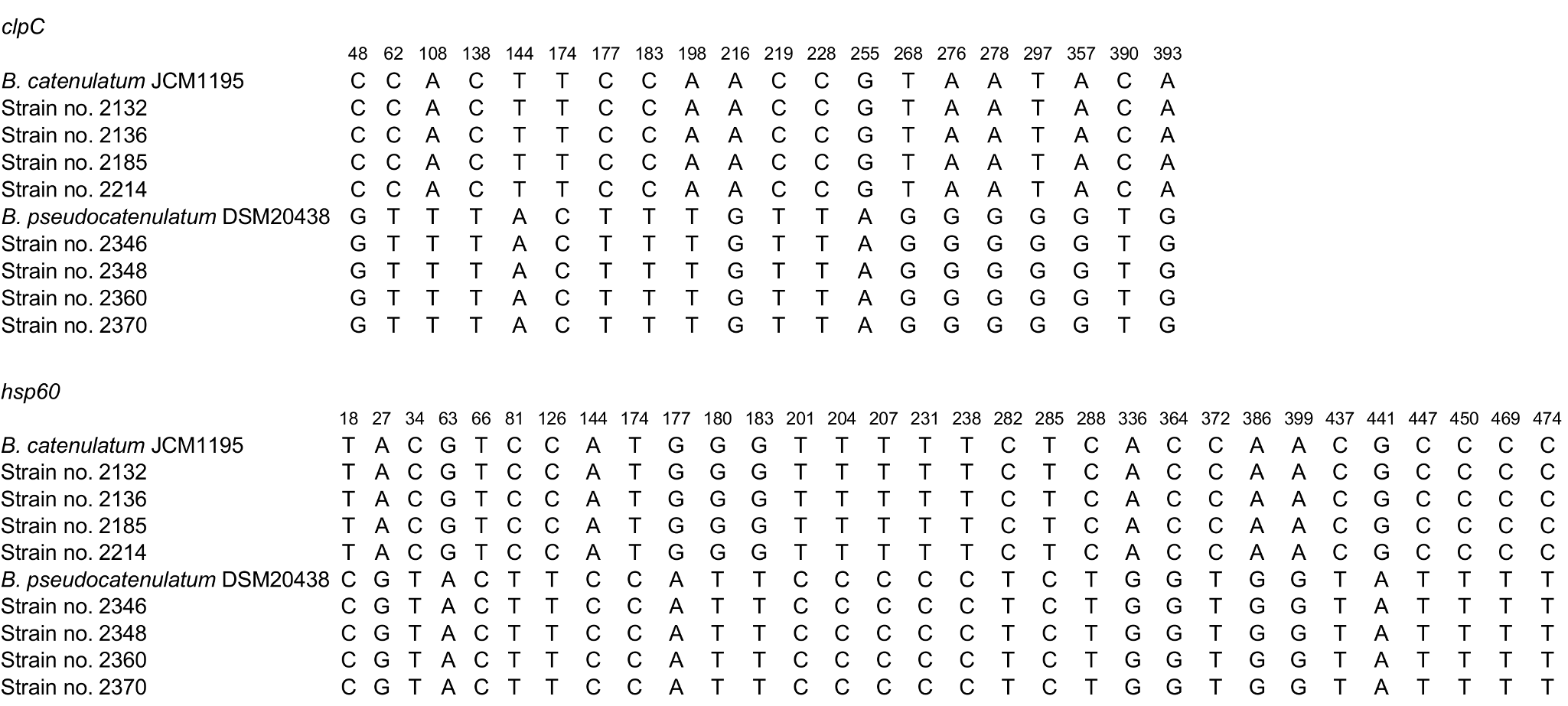

Supplement: Figure S1 — Polymorphic sites among the 24 B. catenulatum/B. pseudocatenulatum continuum at gene clpC and hsp60 . For each gene, all discovered alleles were compared, and only polymorphic sites are shown. Numbering starts at the beginning of the aligned sequence portion of each gene. (TIF) [file pone.0078331.s001.tif]

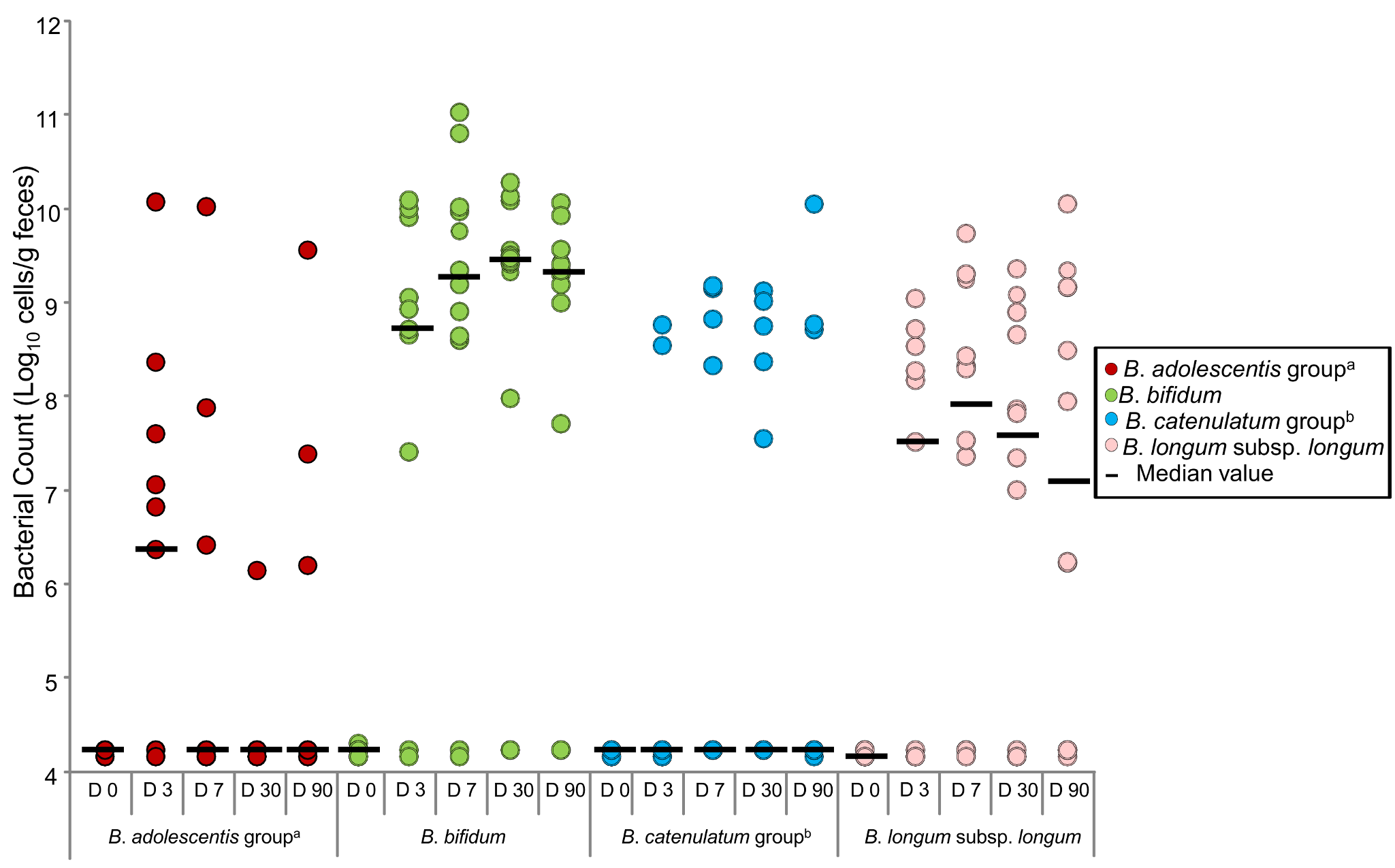

Supplement: Figure S2 — Bifidobacterial counts of each species in infant's feces from 0 to 90 days of age. Bars represent the median values respectively. For values less than the qPCR detection limit (106 cells/g of feces), “detection limit/√2” [34] was used as a complement value. a The B. adolescentis group consists of B. adolescentis genotypes A and B. b The B. catenulatum group consists of B. catenulatum and B. pseudocatenulatum. (TIF) [file pone.0078331.s002.tif]
