# Supplementary material for: Mother-to-Infant Transmission of Intestinal Bifidobacterial Strains Has an Impact on the Early Development of Vaginally Delivered Infant's Microbiota
Source: PLoS One. 2013 Nov 14;8(11):e78331. doi: 10.1371/journal.pone.0078331 (PMC3828338; doi:10.1371/journal.pone.0078331)
Supplement: Table S1 — Information of the infant (gender, gestational ages, weights, place of birth, delivery date). Mother no. 5 gave birth to twins (A, B). (DOCX) [file pone.0078331.s003.docx]

**Table S1**  Information of the infant (gender, gestational ages, weights, place of birth, delivery date).

| **Mode of delivery** | **Infant no.** | **Gender (M/F)** | **Gestational age (week)** | **Weight (g)** | **Place of birth** | **Delivery date** |
| --- | --- | --- | --- | --- | --- | --- |
| Vaginal delivery | 1 | F | 40 | 3665 | Hospital A | 2009-09-25 |
|  | 2 | F | 41 | 3300 | Hospital A | 2009-09-08 |
|  | 3 | F | 37 | 3140 | Hospital A | 2009-08-18 |
|  | 4 | M | 38 | 3030 | Hospital A | 2010-01-28 |
|  | 5(A) | M | 37 | 3120 | Hospital B | 2009-12-11 |
|  | 5(B) | M | 37 | 3040 | Hospital B | 2009-12-11 |
|  | 6 | M | 40 | 3890 | Hospital A | 2010-03-08 |
|  | 7 | F | 42 | 4500 | Hospital C | 2010-03-07 |
|  | 8 | M | 39 | 3790 | Hospital B | 2010-04-02 |
|  | 9 | M | 39 | 3530 | Hospital A | 2010-05-27 |
|  | 10 | M | 39 | 2720 | Hospital B | 2010-05-05 |
|  | 11 | M | 42 | 4050 | Home | 2010-08-03 |
| Cesarean delivery | 12 | F | 39 | 3650 | Hospital A | 2009-09-21 |
|  | 13 | F | 41 | 2980 | Hospital A | 2009-10-22 |
|  | 14 | M | 39 | 2965 | Hospital C | 2009-09-30 |
|  | 15 | F | 40 | 3950 | Hospital A | 2009-11-09 |
|  | 16 | M | 39 | 3395 | Home | 2009-12-23 |

Mother no.5 gave birth to twins (A, B).
