# Supplementary material for: Mother-to-Infant Transmission of Intestinal Bifidobacterial Strains Has an Impact on the Early Development of Vaginally Delivered Infant's Microbiota
Source: PLoS One. 2013 Nov 14;8(11):e78331. doi: 10.1371/journal.pone.0078331 (PMC3828338; doi:10.1371/journal.pone.0078331)
Supplement: Table S2 — Scheme for mother's fecal samples. Samples of feces from mothers were collected twice (at least 1 week apart) before delivery. (DOCX) [file pone.0078331.s004.docx]

**Table S2**  Scheme for mother’s fecal samples. Samples of feces from mothers were collected twice (at least 1 week apart) before delivery.

|  |  | **Days before delivery** | |
| --- | --- | --- | --- |
| **Mode of delivery** | **Mother no.** | **First sample** | **Second sample** |
| Vaginal delivery | 1 | 63 | 51 |
|  | 2 | 53 | 44 |
|  | 3 | 21 | 14 |
|  | 4 | 51 | 44 |
|  | 5 | 17 | 10 |
|  | 6 | 28 | 21 |
|  | 7 | 42 | 36 |
|  | 8 | 38 | 30 |
|  | 9 | 16 | 8 |
|  | 10 | 40 | 33 |
|  | 11 | 64 | 54 |
| Cesarean delivery | 12 | 14 | 7 |
|  | 13 | 37 | 14 |
|  | 14 | 20 | 13 |
|  | 15 | 40 | 33 |
|  | 16 | 40 | 28 |
