# Supplementary material for: Mother-to-Infant Transmission of Intestinal Bifidobacterial Strains Has an Impact on the Early Development of Vaginally Delivered Infant's Microbiota
Source: PLoS One. 2013 Nov 14;8(11):e78331. doi: 10.1371/journal.pone.0078331 (PMC3828338; doi:10.1371/journal.pone.0078331)
Supplement: Table S3 — Numbers of Bifidobacterium strains isolated from each family and submitted for MLST analysis. We used Bifidobacterium strains from 16 families (vaginal delivery: 11 families; Cesarean delivery: 5 families), isolated from mother's and infant's feces at different sampling time points. “-” represents no strains were isolated. Mother's fecal samples were collected twice before delivery (see table S2). Mother no. 5 gave birth to twins (A, B). (DOCX) [file pone.0078331.s005.docx]

**Table S3**  Numbers of *Bifidobacterium* strains isolated from each family and submitted for MLST analysis**.**

| **Mode of delivery** | **Species** | **Family No.** | **First** | **Second** | **Day 0** | **Day 3** | **Day 7** | **Day 30** | **Day 90** | **Total** |
| --- | --- | --- | --- | --- | --- | --- | --- | --- | --- | --- |
| Vaginal delivery | *B*. *adolescentis* | 3 | 8 | 6 | - | 3 | - | - | - | 17 |
|  |  | 5(B) | 3 | 3 | - | 3 | 3 | - | - | 12 |
|  |  | 10 | 2 | - | - | 1 | - | - | - | 3 |
|  | *B*. *bifidum* | 1 | - | 2 | - | - | - | 3 | - | 5 |
|  |  | 2 | 1 | 1 | - | 6 | 8 | 2 | - | 18 |
|  |  | 5(A) | 1 | 1 | - | 3 | 6 | - | - | 19 |
|  |  | 5(B) |  |  | - | 2 | 6 | - | - |  |
|  |  | 6 | 2 | - | - | 1 | - | - | - | 3 |
|  |  | 7 | - | 1 | - | - | - | - | 2 | 3 |
|  |  | 8 | 1 | 2 | - | 3 | 2 | - | 1 | 9 |
|  |  | 11 | 2 | 3 | - | 2 | - | - | - | 7 |
|  | *B*. *catenulatum* | 4 | - | 6 | - | - | - | - | 2 | 8 |
|  |  | 6 | 1 | - | - | 3 | 3 | - | - | 7 |
|  | *B*. *longum* subsp. *longum* | 3 | 6 | 4 | 4 | 6 | 6 | 8 | 8 | 42 |
|  |  | 8 | 3 | 2 | - | 3 | 4 | - | 2 | 14 |
|  |  | 9 | 4 | 3 | - | - | - | 1 | - | 8 |
|  |  | 10 | 2 | - | - | 2 | - | 3 | - | 7 |
|  |  | 11 | 4 | - | - | 4 | 6 | 6 | - | 20 |
|  | *B*. *pseudocatenulatum* | 7 | - | 1 | - | - | 2 | 3 | - | 6 |
|  |  | 9 | 2 | - | - | - | 1 | - | - | 3 |
|  | Total | | 42 | 35 | 4 | 42 | 47 | 26 | 15 | 211 |
| Cesarean delivery | *B*. *longum* subsp. *longum* | **12** | 5 | 3 | - | - | 8 | 6 | 6 | 28 |
|  |  | 13 | 2 | - | - | - | - | 3 | 1 | 6 |
|  |  | 14 | 4 | 5 | - | - | - | - | 3 | 12 |
|  |  | 15 | 2 | - | - | - | - | - | 1 | 3 |
|  |  | 16 | - | 7 | - | - | - | 6 | - | 13 |
|  | Total | | 13 | 15 | 0 | 0 | 8 | 15 | 11 | 62 |

We used *Bifidobacterium* strains from 16 families (vaginal delivery: 11 families; Cesarean delivery: 5 families), isolated from mother’s and infant’s feces at different sampling time points. “－” represents no strains were isolated. Mother’s fecal samples were collected twice before delivery (see table S2). Mother no.5 gave birth to twins (A, B).
